# Polar Order Evolutions near the Rhombohedral to Pseudocubic and Tetragonal to Pseudocubic Phase Boundaries of the BiFeO_3_-BaTiO_3_ System

**DOI:** 10.3390/ma8125462

**Published:** 2015-12-02

**Authors:** Yongxing Wei, Changqing Jin, Yiming Zeng, Xiaotao Wang, Gang Xu, Xiaoli Wang

**Affiliations:** 1 School of Materials and Chemical Engineering, Xi’an Technological University, Xi’an 710021, China; eaglejin@xatu.edu.cn (C.J.); xugang@xatu.edu.cn (G.X.); 2 Sate Key Laboratory of Advanced Technologies for Comprehensive Utilization of Platinum Metals, Kunming Institute of Precious Metals, Kunming 650106, China; zym@ipm.com.cn; 3 MOE Key Laboratory for Non-equilibrium Synthesis and Modulation of Condensed Matter, School of Science, Xi’an Jiaotong University, Xi’an 710049, China; In.01211@stu.xjtu.edu.cn (X.W.); xlwang1@mail.xjtu.edu.cn (X.W.)

**Keywords:** morphotropic phase boundary, piezoelectric, BiFeO_3_

## Abstract

Solid solutions of (1-*x*)BiFeO_3_-*x*BaTiO_3_ (BF-BT, 0.05 ≤ *x* ≤ 0.98) were prepared and characterized. It was found that the dielectric constant ε*_m_*, remnant polarization *P_r_* and piezoelectric coefficient *d*_33_ reach their maximum values near the rhombohedral–pseudocubic phase boundary. In particular, the 0.7BF-0.3BT composition shows large polarization (*P_r_* > 20 μC/cm^2^) and a temperature-stable piezoelectric property (*d*_33_ > 100 pC/N when the annealing temperature is lower than ~400 °C). Near the tetragonal–pseudocubic phase boundary, ε*_m_* and *P_r_* decrease, and the piezoelectric property vanishes when the BF content reaches 4 mol %.

## 1. Introduction

Bi-based perovskite materials, BiMeO_3_ (BM), in which Me can be a single trivalent cation or a mixture of two cations, have been studied because of their high polarization and *T_c_* values. However, synthesis of a single-phase BM perovskite ceramic material by conventional synthesized methods is challenging; this may be due to the small radius of the Bi cation [1,2,3,4]. The addition of PbTiO_3_ (PT) or BaTiO_3_ (BT) with large A-site ions into BM ceramics to form solid solutions can effectively stabilize the perovskite structure. Some BM-PT systems exhibit a large *d*_33_ piezoelectric coefficient with a high *T_c_* near the morphotropic phase boundary (MPB) [5,6,7,8]. In particular, the piezoelectric activity in 0.36BiScO_3_-0.64PbTiO_3_ (*d*_33_ > 460 PC/N, *T_c_* = 450 °C) is comparable to that in the Pb(Zr_1-*x*_Ti*_x_*)O_3_ (PZT) material [5]. On the other hand, for BM-BT systems, even the end members are both ferroelectrics with low symmetry structures (Bi(Ti_1/2_Zn_1/2_)O_3_-BaTiO_3_, BiAlO_3_-BaTiO_3_, *etc.*), and the decrease in BT content leads to the appearance of the undesired pseudo-cubic phase. The composition-driven ferroelectric-relaxor transition is usually found near the phase boundary between the tetragonal (T) and pseudocubic (PC) phases [9,10,11]. On the BM side of the phase diagram, limited solubility gives rise to difficulty in studying the structures and electrical properties of these materials.

BiFeO_3_-BaTiO_3_ (BF-BT) was first investigated for its multiferroic properties [12,13,14]. A small amount of BT can effectively stabilize the perovskite phase [14]. Earlier investigations of the BF-BT system have shown that the system undergoes structural transitions, from the rhombohedral (R) to the cubic (C) and to the T phase, with the addition of BT [12]. The observation of ferroelectric behavior in compositions with a BT content of 33 to 50 mol % suggests that the intermediate phases might be considered to have PC symmetry [15,16,17]. Recently, good piezoelectric properties near the rhombohedral–pseudocubic (R–PC) phase boundary have been found in unmodified 0.7BF-0.3BT ceramics (*d*_33_ = 134 pC/N) and Mn-modified 0.725BF-0.275BT ceramics (*d*_33_ = 136 pC/N) [18,19,20]. Near the tetragonal–pseudocubic (T–PC) phase boundary, a smeared dielectric anomaly has been found in 0.04BF-0.96BT ceramics, with a maximum dielectric constant ε*_m_* of ~800, but the related ferroelectric and piezoelectric properties have rarely been reported [21,22].

To understand the polar order mechanism of BF-BT, systematic investigations of electrical properties in the BF-BT system should be carried out. In this work, (1-*x*)BF-*x*BT (0.05 ≤ *x* ≤ 0.98) solid solution ceramics were prepared. The different polarization behaviors near the R-PC and T-PC phase boundaries were reported and discussed.

## 2. Experimental Methods

The (1-*x*)BF-*x*BT (0.05 ≤ *x* ≤ 0.98) solid solution ceramics were prepared using the mixed oxide method. The starting reagents of Bi_2_O_3_ (>99%) (Sinapharm Chemical Reagent Co., Ltd, Shanghai, China), Fe_2_O_3_ (>99%) (Sinapharm Chemical Reagent Co., Ltd.), BaCO_3_ (>99%) (Sinapharm Chemical Reagent Co., Ltd.) and TiO_2_ (>99%)(Sinapharm Chemical Reagent Co., Ltd.) were carefully weighed in stoichiometric ratios and wet mixed thoroughly by ball-milling for 10 h. The mixture was then dried and calcined at 750–1140 °C (Table 1) for 2 h in a cove red corundum crucible. The calcined powder was ball-milled and dried. Pellets with a diameter and thickness of 12 and 1–2 mm, respectively, were pressed using a 10% polyvinyl alcohol binder. The pellets were sintered in a covered corundum crucible at 930–1340 °C (Table 1) for 3 h. To reduce the volatilization of bismuth oxide during sintering, the pellets were buried with the mixture of Bi_2_O_3_ and calcined ZrO_2_. For X-ray diffraction measurements, the ceramics were crushed into fine powders. For electric measurements, silver paste was painted onto both sides of the ground ceramic pellets and fired at 810 °C for 10 min. Prior to the piezoelectric measurements, the ceramics were poled under 35–70 kV/cm in silicone oil for 10 min at room temperature.

**Table 1:** Calcination and sintering temperatures of (1-*x*)BiFeO_3_-BaTiO_3_ ((1-*x*)BF-*x*BT) system.

Compositions	Temperature (°C)	
	Calcination	Sintering
0.95BF-0.05BT	750	930
0.9BF-0.1BT	750	960
0.8BF-0.2BT	750	980
0.75BF-0.25BT	800	990
0.7BF-0.3BT	800	1000
0.65BF-0.35BT	800	1050
0.6BF-0.4BT	800	1050
0.55BF-0.45BT	850	1090
0.5BF-0.5BT	900	1100
0.4BF-0.6BT	950	1140
0.2BF-0.8BT	1000	1220
0.1BF-0.9BT	1080	1260
0.04BF-0.96BT	1140	1340
0.02BF-0.98BT	1140	1340

Crystal structures of (1-*x*)BF-*x*BT ceramics were detected using an X-ray diffractometer (XRD, Bruker AXS D8 ADVAMDMCE, Karlsruhe, German). The lattice parameters were refined based on XRD patterns using the Rietveld method, as implemented in the Topas 4.2 software. The space groups for compositions with 0.1 ≤ *x* ≤ 0.3, 0.3 < *x* ≤ 0.9 and 0.9 < *x* ≤ 0.98 were chosen as rhombohedral R3c, cubic Pm $\bar{3}$m, and tetragonal P4mm, respectively. The dielectric measurements were performed using an automated system, with a temperature-controlled sample chamber and an Agilent 4284A LCR meter controlled by a personal computer. The ferroelectric hysteresis loop was measured using an automatic ferroelectric test system [23]. The applied electric field signal is sinusoidal with the frequency of 10 Hz. The *d*_33_ values were measured using a piezo-*d*_33_meter (ZJ-3AN; Institute of Acoustics, Beijing, China).

Structural and dielectric measurements of unpoled and poled ceramics for 0.7BF-0.3BT, 0.65BF-0.35BT and 0.6BF-0.4BT were performed. The ceramics were poled for 10 min at room temperature. Structural and dielectric measurements of poled ceramics were carried out after 24 h. Both the unpoled and poled ceramics were polished for structural measurements.

## 3. Results and Discussion

The diffraction peaks of all of the compositions could be indexed as a single perovskite structure, with no observed impure phase (see the Supplementary Information).

### 3.1. Structural and Electrical Properties near the R-PC Phase Boundary

**Figure 1:**
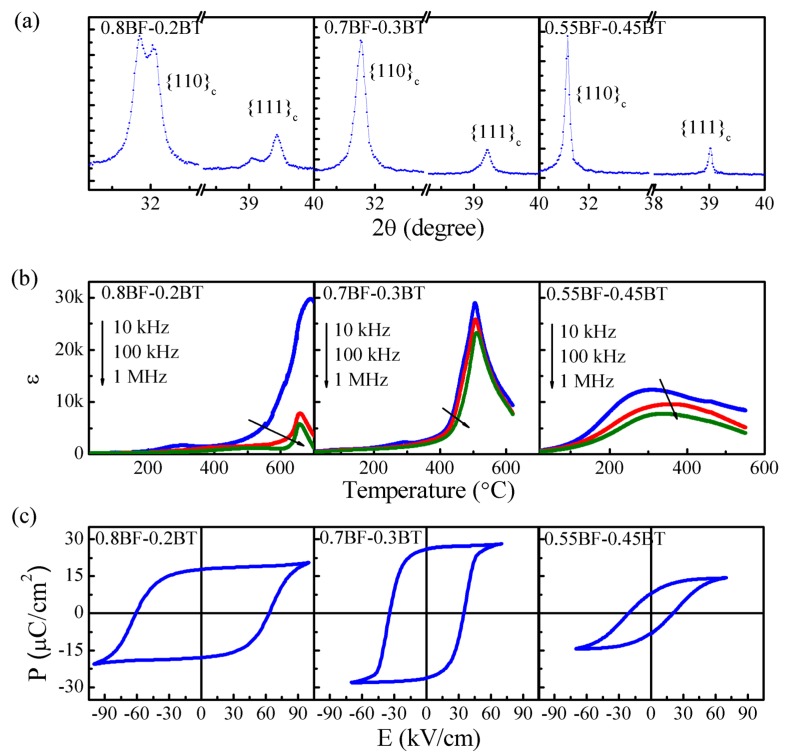
Structural and electrical properties of the BF-BT system near the BF side. (**a**) X-ray diffractometer (XRD) patterns; (**b**) temperature dependence of the dielectric constant ɛ (1 MHz); and (**c**) polarization hysteresis curves of 0.8BF-0.2BT, 0.7BF-0.3BT and 0.55BF-0.35BT ceramics.

Figure 1 shows the XRD patterns, temperature dependences of the dielectric constant ε, and polarization hysteresis loops for 0.8BF-0.2BT, 0.7BF-0.3BT and 0.55BF-0.45BT ceramics. According to the splitting of {110}_c_ and {111}_c_ reflections (Figure 1a), the 0.8BF-0.2BT ceramic most likely exhibits a highly distorted rhombohedral structure, while the 0.7BF-0.3BT ceramic shows a weak rhombohedral distortion. No clear splitting of {110}_c_ and {111} reflections was found in the compositions with a BT content larger than 30 mol %.

**Figure 2:**
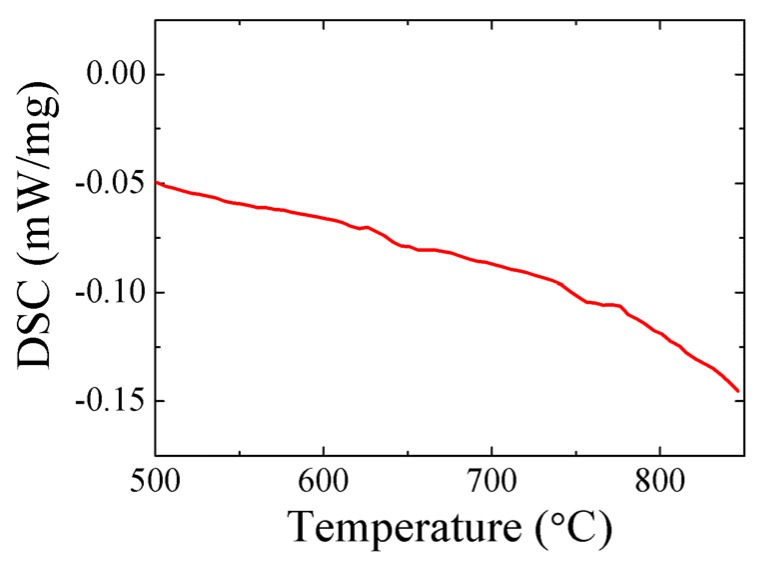
Differential scanning calorimetry (DSC) curve of 0.8BF-0.2BT.

Figure 1b shows that the 0.8BF-0.2BT ceramic exhibits a sharp dielectric peak at ~657 °C. The temperature *T_m_* for the dielectric peak is frequency-independent. However, no endothermic or exothermic peak could be found near *T_m_* (Figure 2). Furthermore, the temperature dependence of the structures also indicates the coexistence of the rhombohedral and cubic structures over a 100 °C range around *T_m_* [35]. It may therefore be reasonable to suggest that 0.8BF-0.2BT is ferroelectric with the diffuse phase transition. The dielectric constant of 0.7BF-0.3BT increases abruptly for temperature higher than 400 °C. The dielectric anomaly is broad, with a maximum value ε*_m_* > 20,000. The corresponding *T_m_* is frequency-dependent. This suggests that the high-temperature dielectric behavior of 0.7BF-0.3BT may be relaxor-like. The 0.55BF-0.45BT ceramic displays a frequency-dependent and very diffuse dielectric anomaly, with a ε*_m_* of 7780 at 1MHz, and the dielectric constant above *T_m_* decreases slowly with increasing temperature. Accordingly, the increasing level of BT substitution leads to a tendency to increase the structural disorder and increase the diffuseness of the dielectric anomaly.

The 0.8BF-0.2BT and 0.7BF-0.3BT ceramics show normal polarization switching behavior at room temperature (Figure 1c). Considering the dielectric and ferroelectric data, it is possible that 0.7BF-0.3BT displays a temperature-driven ferroelectric-relaxor transition. A slim ferroelectric hysteresis loop was found for the 0.55BF-0.45BT ceramic, possibly indicating the presence of the relaxor state.

**Figure 3:**
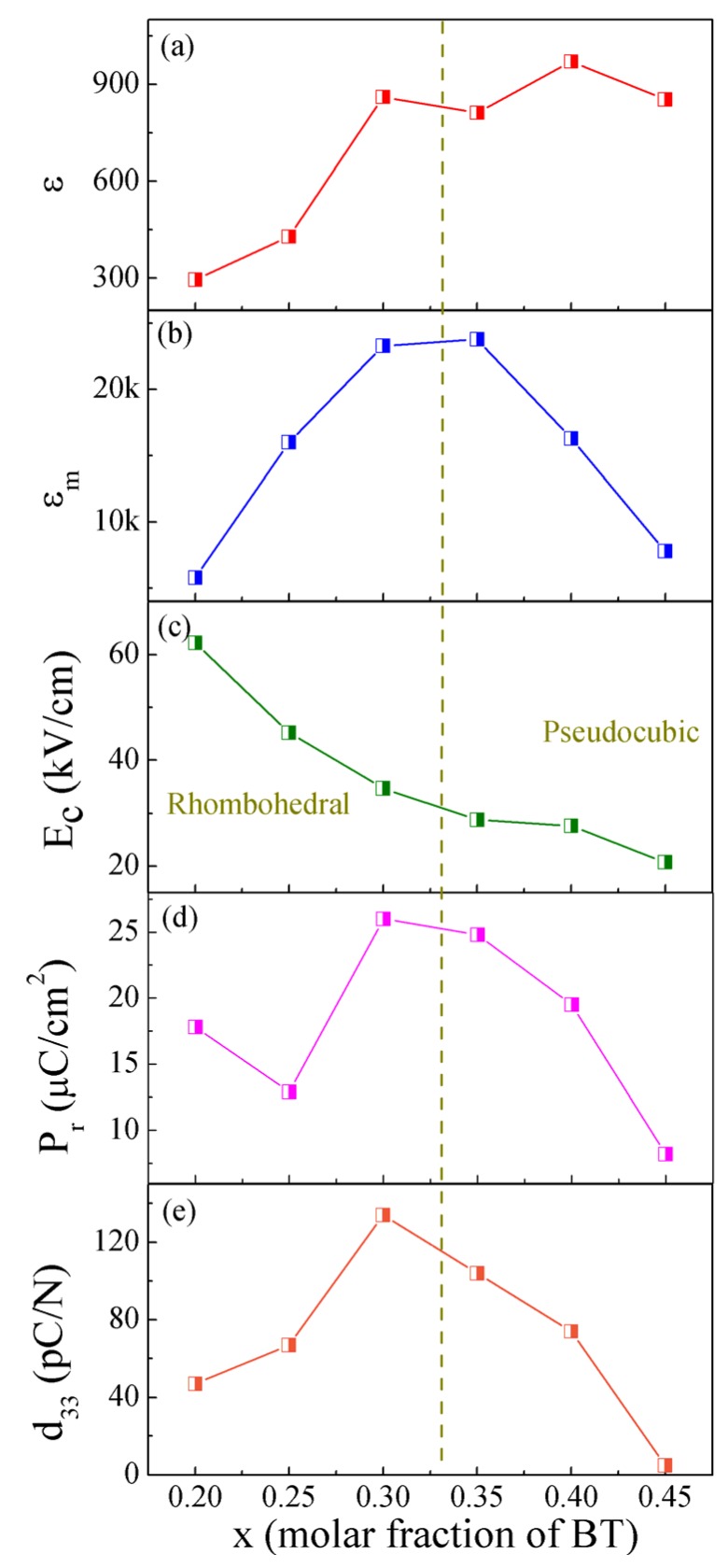
(**a**) Room-temperature ɛ; (**b**) maximum dielectric constant ɛ_m_; (**c**) coercive field *E_c_*; (**d**) remnantpolarization *P_r_*; and (**e**) piezoelectric coefficient *d*_33_ as a function of the BT content for the BF-BT system.

The composition dependences of the dielectric, ferroelectric, and piezoelectric properties for the BF-BT system are shown in Figure 3. For compositions with rhombohedral distortions, the values of room-temperature ε and ε*_m_* increase with increasing BT content (Figure 3a,b). The largest ε*_m_* is found for the composition near the phase boundary. Due to the large *E_c_*, 0.8BF-0.2BT and 0.75BF-0.25BT ceramics were not poled to saturation. This means that the measured *P_r_* may be lower than the intrinsic values. The addition of BT induces decreases in *E_c_*, confirming the transition from hard ferroelectrics to soft ferroelectrics (Figure 3c) [18,19]. The 0.7BF-0.3BT ceramic exhibits optimum ferroelectric and piezoelectric properties (Figure 3d,e). For compositions with a pseudo-cubic structure, the values of ε*_m_*, *P_r_* and *d*_33_ all decrease with the increasing BT content.

Table 2 lists the ferroelectric and piezoelectric parameters for the 0.7BF-0.3BT system, pure BF ceramic, and other BF-based systems [1,24,25,26,27,28,29,30,31,32,33,34]. The pure BF ceramic exhibits a very high *T_c_* and *d*_33_ of 44 pC/N [1,25]. Introducing rare earth ions with the same valence at the BF A-site does not result in an enhancement of the piezoelectric property [25,26,27,28,29]. This may be caused by the large *E_c_*. Solid solution systems of BF-BT, BF-PT and BiFeO_3_-(Bi_0.5_K_0.5_)TiO_3_ contain two cations with different valences on both the A-site and B-site. However, the value of *d*_33_ for the BiFeO_3_-PbTiO_3_ and BiFeO_3_-(Bi_0.5_K_0.5_)TiO_3_ systems is not higher than 100 pC/N [30,31]. Excellent piezoelectric performance is found for compositions of the modified BF-PT systems [32,34]. Interestingly, the compositions of the modified BF-PT systems and 0.7BF-0.3BT all have weak ferroelectric distortion, with a relatively low *E_c_*.

**Table 2:** Ferroelectric and piezoelectric properties of 0.7BF-0.3BT, BiFeO_3_ (BF) and other BF-based ceramics.

Compositions	*P_r_* (μC/cm^2^)	*E_c_* (kV/cm)	*d*_33_ (pC/N)	T_c_/T_m_ (°C)	References
0.7BF-0.3BT	26.0	34.6	134	511	-
BF	~13	>50	44	830~850	[1,24]
Bi_0.92_Dy_0.08_FeO_3_	25.2	>100	37	-	[25]
Bi_0.875_Sm_0.125_FeO_3_	15.1	>80	29	-	[26]
Bi_0.91_La_0.05_Tb_0.04_FeO_3_	-	-	9	-	[27]
Bi_0.85_La_0.15_FeO_3_	12.1	>60	28	-	[28]
(Bi_1-*x*_Nd*_x_*)FeO_3_(*x* = 0–0.15)	-	-	26~28	-	[29]
0.675BiFeO_3_-0.325PbTiO_3_	62	>40	85	642	[30]
0.6BiFeO_3_-0.4(Bi_0.5_K_0.5_)TiO_3_	~15	~40	37	441	[31]
0.57(Bi_0.8_La_0.2_)(Ga_0.05_Fe_0.95_)O_3_-0.43PbTiO_3_	~30	~20	295	264	[32]
0.511BiFeO_3_-0.326PbZrO_3_-0.163PbTiO_3_	~12	~40	101	431	[33]
0.33BiFeO_3_-0.2BaZrO_3_-0.47PbTiO_3_	~30	~20	~270	270	[34]

**Figure 4:**
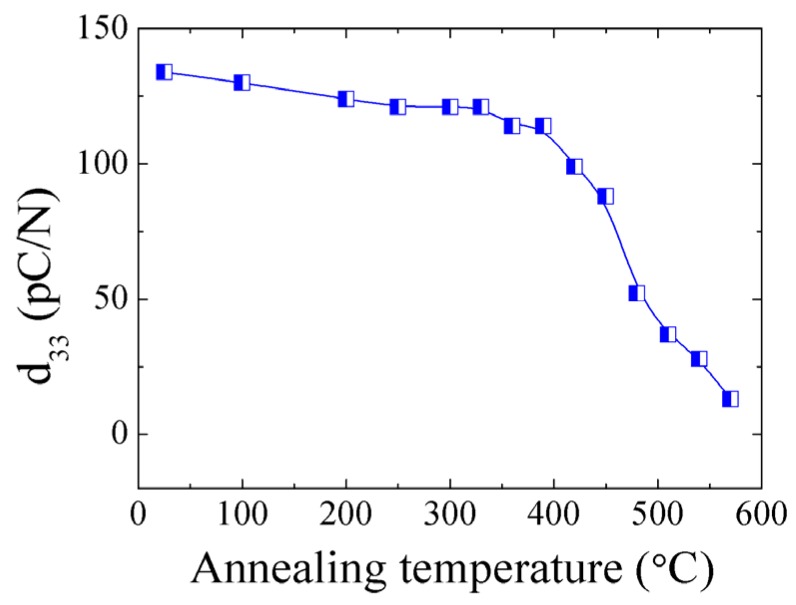
Annealing temperature dependence of room-temperature *d*_33_ for 0.7BF-0.3BT.

The annealing temperature dependence of *d*_33_ for 0.7BF-0.3BT is shown in Figure 4. The *d*_33_ was measured after the sample was annealed at selected temperatures for one hour. The value of *d*_33_ is stable with respect to temperatures below ~400 °C (>100 pC/N) and then drops noticeably around the depolarization temperature *T_d_*. The *T_d_* of 0.7BF-0.3BT is ~420 °C, which is much higher than that of the lead-free (Bi_0.5_Na_0.5_)TiO_3_-based ceramics [36].

**Figure 5:**
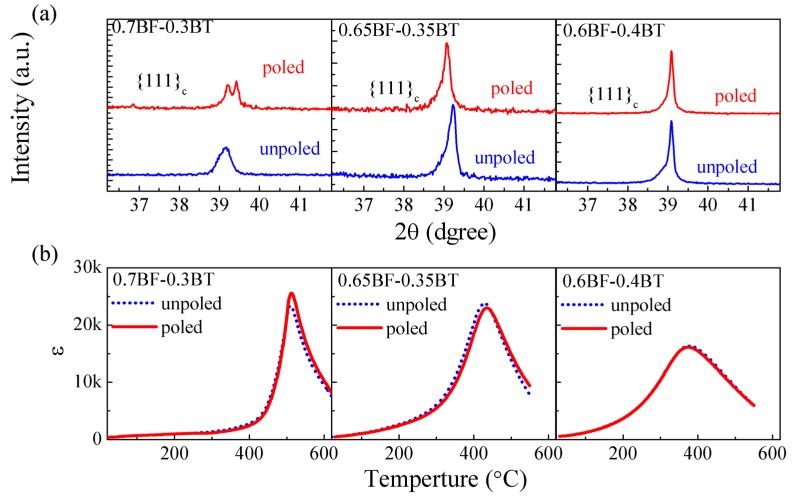
(**a**) XRD patterns; (**b**) Temperature dependence of the dielectric constant ɛ of unpoled and poled samples for BF-BT compositions across the rhombohedral-pseudocubic phase boundary.

Figure 5 shows the XRD patterns and the temperature dependence of ε for unpoled and poled BF-BT ceramics near the R-PC phase boundary. Clear splits of {111}_c_ reflection were found in 0.7BF-0.3BT after poling. This suggests that the electric field triggers an obvious modification of the lattice parameters in 0.7BF-0.3BT. However, the crystal symmetry does not change after poling. This shows that the electric-field-induced phenomenon in 0.7BF-0.3BT may be different from that in MPB-based system [37,38]. The possible polarization directions (<111>_c_ directions) could be only related to the rhombohedral distortion. Furthermore, the value of ε_m_ for the poled 0.7BF-0.3BT is much larger than that for the unpoled sample (Figure 5b). On the other hand, for 0.65BF-0.35BT and 0.6BF-0.4BT, there are no obvious differences in the structure and ε_m_ before and after poling. The detailed structure data are shown in the Supplemental Information.

### 3.2. Structural and Electrical Properties near the T-PC Phase Boundary

**Figure 6:**
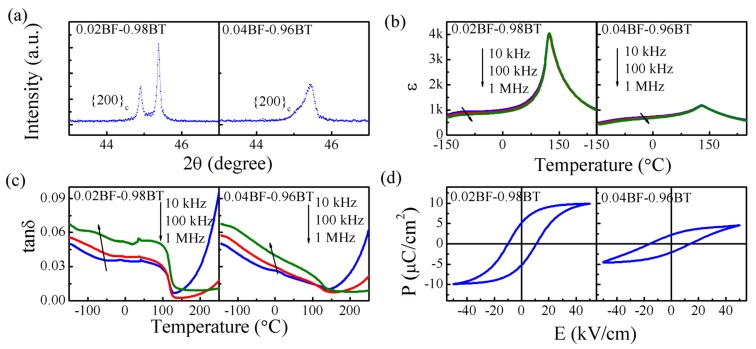
Structural and electrical properties of the BF-BT system near the BT side. (**a**) XRD patterns; (**b**) temperature dependence of dielectric constant ɛ; (**c**) temperature dependence of dielectric loss tanδ; and (**d**) polarization hysteresis curves of 0.02BF-0.98BT and 0.04BF-0.96BT ceramics.

The XRD patterns, temperature dependences of dielectric properties, and polarization hysteresis loops of 0.02BF-0.98BT and 0.04BF-0.96BT ceramics are shown in Figure 6. Clear splitting of the {200}_c_ peak reflections for the 0.02BF-0.98BT ceramic reveals the presence of a tetragonal distortion (Figure 6a). However, for the 0.04BF-0.96BT ceramic, the splitting of {200}_c_ reflections is much weaker. This suggests that the tetragonal distortions are effectively suppressed when BF content reaches 4 mol %.

The 0.02BF-0.98BT ceramic exhibits a sharp dielectric peak near 123 °C, with a ε*_m_* of 4300 (Figure 6b). The corresponding *T_m_* is frequency-independent. The sharp dielectric peak can be related to the transition from the ferroelectric T phase to the paraelectric C phase. Additionally, 0.02BF-0.98BT displays another weak and broad dielectric anomaly around −130 °C. As is known, pure BT ceramic has two dielectric anomalies below room temperature that are related to the tetragonal-orthorhombic and orthorhombic-rhombohedral phase transitions. The low temperature dielectric behavior of 0.02BF-0.98BT suggests that a small amount of BF content weakens the low-temperature phase transitions of BT. The dielectric anomaly of 0.04BF-0.96BT is smeared (ε*_m_* = 1170), with a frequency-independent *T_m_*. The tanδ value for 0.02BF-0.98BT decreases abruptly near *T_m_* (Figure 6c). On the other hand, the decrease in tanδ near *T_m_* for 0.04BF-0.96BT is gradual. The dielectric data of 0.04BF-0.96BT may suggest the diffused phase transition [21].

The ferroelectric behaviors of 0.02BF-0.98BT and 0.04BF-0.96BT ceramics are also different (Figure 6d). A normal ferroelectric hysteresis loop is observed in the former, with a *P_r_* of 5.3 μC/cm^2^, while a slim hysteresis loop is found in the latter, with a *P_r_* of 2.2 μC/cm^2^. The value of *d*_33_ for 0.02BF-0.98BT is 14 pC/N. However, *d*_33_ vanishes for 0.04BF-0.96BT. The evolutions of the dielectric, ferroelectric, and piezoelectric properties near the BT end member reveal that long-range ferroelectric order is destroyed when the BF content is 4 mol %.

### 3.3. Composition Dependence of Lattice Parameters

**Figure 7:**
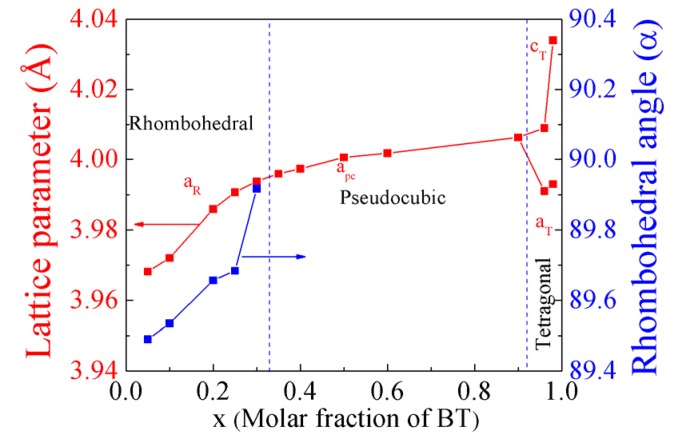
Lattice parameters of the BF-BT system as a function of the BT content.

Figure 7 shows the lattice parameters of BF-BT ceramics as a function of the BT content. The ferroelectric distortion near the BT side is much more easily disrupted than that near the BF side. In the tetragonal region, the *c*_T_ value for 0.04BF-0.96BT (4.009 Å) decreased significantly relative to that for 0.02BF-0.98BT (4.034 Å), while the *a*_T_ values of the two compositions are similar. The reduction of the c axis leads to the suppression of the tetragonal distortion. The calculated lattice parameters for the rhombohedral BF-BT compositions are described in the hexagonal unit cell with the R3c space group. To clearly observe the rhombohedral distortion, the lattice parameters in the perovskite-type unit cell are used. The transformation relation could be expressed as:

(1) $$a_{r}=\frac{1}{3}\sqrt{3{a_{\text{H}}}^{2}+{\left(\frac{c_{\text{H}}}{2}\right)}^{2}}$$

(2) $$\text{sin}\frac{\alpha }{2}=\frac{3}{2}\frac{1}{\sqrt{3+{\left(\frac{c_{\text{H}}}{2a_{\text{H}}}\right)}^{2}}}$$

where *a*_H_ and *c*_H_ are the lattice parameters in the hexagonal unit cell and *a*_r_ and α are the lattice parameter and rhombohedral angle in the perovskite-type unit cell, respectively. The values of α and *a*_R_ both increase gradually with the addition of BT. When the BT content is 25 mol %, the composition still shows a large rhombohedral distortion (α = 89.68°).

### 3.4. Possible Reasons for the Different Behaviors near the Two Phase Boundaries

It is known that both temperature-driven polymorphic structure transitions (e.g., BT-based ceramics, (K*_x_*Na_1-*x*_)NbO_3_ based ceramics) and composition-driven MPB (e.g., Pb(Zr*_x_*Ti_1-*x*_)O_3_, Pb(Mg_1/3_Nb_2/3_)O_3_-PbTiO_3_) can improve the piezoelectric activity [36,39,40,41,42]. Although most MPBs are related to the transitions between the R phase and T phase, the composition-driven R-C or T-C transition may also lead to the enhancement in electrical properties [39]. The phase boundary (separation of the R phase and PC phase) for the BF-BT system could be regarded as MPB. Near the MPB, the BF-BT system shows a temperature-driven and composition-driven ferroelectric-relaxor transition, which is similar to that in Pb(Mg_1/3_Nb_2/3_)O_3_-PbTiO_3_ [41]. However, no clear ferroelectric distortions could be found in the unpoled compositions that show the enhanced electrical properties.

Near the T-PC phase boundary, when the content of BF is only 4 mol %, the tetragonal distortions and long-range ferroelectric order are effectively suppressed. The electrical behaviors near the two phase boundaries in the BF-BT system are quite different. The mixtures of atoms with different electronegativities, valences, and radii both on the A-site and B-site, coupled with the structural inhomogeneity, increase the complexity of the structure-property relation. However, two important factors should be considered.

First, the Bi(6s)–O(2p) covalent bonds are much stronger than the Ti(4d)-O(2p) covalent bonds. The Ti cation displacement in BT along the [001]_c_ direction from the central of ideal cubic perovskite is ~0.14 Å [43]. For BF, the Bi cation displacement along the [111]_c_ direction is ~0.676 Å [44]. This may suggest that the Ti–O covalent bond is more easily weakened than the Bi–O covalent bond.

In addition, the effects of the structure parameters near the BF and BT sides are also different. Near the BF side, the addition of Ba^2+^ ions with a large radius expands the unit cell, as shown by the compositional dependence of the lattice parameters (Figure 7). The displacement space of the Bi^3+^ ions is enlarged. Large off-center displacements could be present in Bi-rich regions. As a result, large polarization is possible near the R-PC phase boundary of the BF-BT system. A similar effect has been reported in the Ba(Ti_1-*x*_Sn*_x_*)O_3_ system, in that the radii of the Sn^4+^ ions are much larger than those of the Ti^4+^ ions [45]. Although there are no reports on the electrical properties near the BM side in other BM-BT systems, a polarization enhancement is expected. Near the BT side, the addition of BF leads to the decrease in the length of c axis parameter. The displacement along the [001]_c_ direction for the Ti^4+^ ions is reduced, as also shown by neutron diffraction data [21]. The similar phenomenon was observed in other BM-BT systems [9,10,11]. The reason for this is still unclear. Nevertheless, the types of A-site ions should be considered. For BM-PT systems that contain ferroelectric active Bi^3+^ and Pb^2+^ cations on the A-site, the behavior is different. The length of the c axis and the tetragonal distortion for BiScO_3_-PbTiO_3_ and Bi(Ti_1/2_Mg_1/2_)O_3_-PbTiO_3_ systems near the PT side decrease slowly with the decreasing PT content [5,7]. In contrast, the addition of BF or Bi(Ti_0.5_Zn_0.5_)O_3_ leads to the increase in the c axis and the tetragonal distortion near the PT region [46,47]. A first-principles theoretical calculation should be carried out to further understand the two phase boundaries for BF-BT.

## 4. Conclusions

In conclusion, the polar order evolutions near the R-PC and T-PC phase boundaries in the BF-BT system are different. Near the R-PC phase boundary, 0.7BF-0.3BT exhibits a large polarization and a temperature-stable piezoelectric property (T_d_ = ~420 °C). The increase of BF content leads to the decrease of the electrical parameters near the BT side. The long-ranged ferroelectric order is disrupted when the BF content is only 4 mol %. An understanding of the electrical behaviors in the BF-BT system may help in the search for high-performance Pb-free piezoelectric ceramics based on similar phase boundaries.

## Supplementary Materials

The following are available online at www.mdpi.com/1996-1944/8/12/5462/s1.
