# Supplementary material for: Polar Order Evolutions near the Rhombohedral to Pseudocubic and Tetragonal to Pseudocubic Phase Boundaries of the BiFeO3-BaTiO3 System
Source: Materials (Basel). 2015 Dec 2;8(12):8355–65. doi: 10.3390/ma8125462 (PMC5458829; doi:10.3390/ma8125462)
Supplement: Supplementary file 1 [file materials-08-05462-s001.pdf]

Supplementary Information

Polar Order Evolutions near the Rhombohedral to Pseudocubic and Tetragonal to Pseudocubic Phase Boundaries of the $BiFeO_3$-$BaTiO_3$ System

Yongxing Wei [1,*], Changqing Jin [1], Yiming Zeng [2], Xiaotao Wang [3], Gang Xu [1] and Xiaoli Wang [3]

The results of X-ray diffraction (XRD) experiment

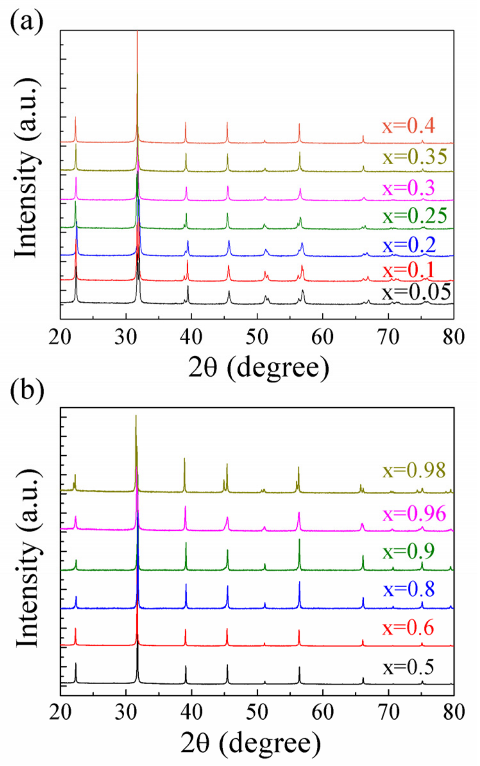

Figure S1. XRD patterns of (1-x)BF-xBT solid solution system with compositions of (**a**) x = 0.05–0.4 and (**b**) 0.5–0.98. All the compositions show the single perovskite structure, without the formation of secondary phase.

The XRD data for the compositions before and after poling

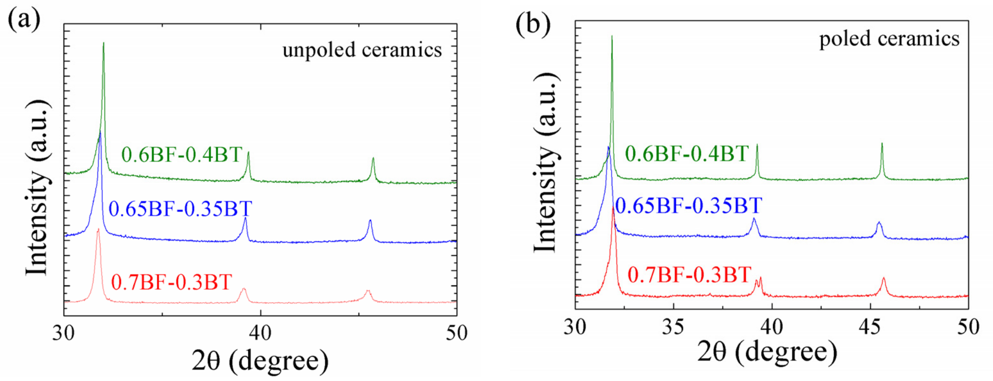

Figure S2. XRD patterns of (1-x)BF-xBT (x = 0.3, 0.35 and 0.4) ceramics (**a**) before poling and (**b**) after poling. The splits of $\{111\}_c$ reflection are clear for 0.7BF-0.3BT ceramic after poling.
